# Correction: Catalytic generation of *ortho*-quinone dimethides *via* donor/donor rhodium carbenes

**DOI:** 10.1039/d4sc90194k

**Published:** 2024-11-05

**Authors:** Mingchun Gao, Jose M. Ruiz, Emily Jimenez, Anna Lo, Croix J. Laconsay, James C. Fettinger, Dean J. Tantillo, Jared T. Shaw

**Affiliations:** a Department of Chemistry, University of California One Shields Avenue Davis California 95616 USA jtshaw@ucdavis.edu

## Abstract

Correction for ‘Catalytic generation of *ortho*-quinone dimethides *via* donor/donor rhodium carbenes’ by Mingchun Gao *et al.*, *Chem. Sci.*, 2023, **14**, 6443–6448, https://doi.org/10.1039/D3SC00734K.

Due to a miscommunication during submission and galley review, Jose M. Ruiz was not originally listed as a co-first author. The author list has been updated to correct this oversight.

The Royal Society of Chemistry apologises for these errors and any consequent inconvenience to authors and readers.

